# Priority Nursing Populations for Mental Health Support Before and During COVID-19: A Survey Study of Individual and Workplace Characteristics

**DOI:** 10.1177/08445621221098833

**Published:** 2022-05-17

**Authors:** Farinaz Havaei, Maura MacPhee, Andy Ma, Yue Mao

**Affiliations:** 1 School of Nursing, 70439 University of British Columbia, Vancouver, Canada; 2 Department of Educational and Counselling Psychology, and Special Education, 8166 University of British Columbia, Vancouver, Canada

**Keywords:** Nursing, mental health, demographic characteristics, workplace characteristics, health authority, logistic models

## Abstract

**Background:**

Nursing is a high-risk profession and nurses’ exposure to workplace risk factors such as heavy workloads and inadequate staffing is well documented. The COVID-19 pandemic has exacerbated nurses’ exposure to workplace risk factors, further deteriorating their mental health. Therefore, it is both timely and important to determine nursing groups in greatest need of mental health interventions and supports.

**Purpose:**

The purpose of this study is to provide a granular examination of the differences in nurse mental health across nurse demographic and workplace characteristics before and after COVID-19 was declared a pandemic.

**Methods:**

This secondary analysis used survey data from two cross-sectional studies with samples (Time 1 study, 5,512 nurses; Time 2, 4,523) recruited from the nursing membership (∼48,000) of the British Columbia nurses’ union. Data was analyzed at each timepoint using descriptive statistics and ordinal logistic regression.

**Results:**

Several demographic and workplace characteristics were found to predict significant differences in the number of positive screenings on measures of poor mental health. Most importantly, in both survey times younger age was a strong predictor of worse mental health, as was full-time employment. Nurse workplace health authority was also a significant predictor of worse mental health.

**Conclusions:**

Structural and psychological strategies must be in place, proactively and preventively, to buffer nurses against workplace challenges that are likely to increase during the COVID-19 crisis.

## Background & purpose

Nursing is a high-risk profession, and nurses’ exposure to workplace risk factors such as human suffering and death, heavy workloads, inadequate staffing, and workplace violence is well documented (MacPhee et al., 2017; Spector, 2021; The Canadian Federation of Nurses’ Unions, 2017). In Canada, the state of nurses’ mental health has been a documented concern of health employers and decision makers for over two decades (Shields & Wilkins, 2006). Nurses’ mental health is especially important given the growing shortage of nurses (Murphy et al., 2012) and empirical evidence linking nurses’ poor mental health with turnover and absenteeism (Hayes et al., 2012) and adverse patient outcomes (Van Bogaert et al., 2013). The COVID-19 pandemic has exacerbated nurses’ exposure to workplace risk factors, further deteriorating their mental health (Havaei et al., 2021b; Havaei et al., 2021c; Smith et al., 2020). Canada is entering the fourth wave of the pandemic and more dangerous COVID-19 variants continue to emerge. The purpose of this study is to provide a granular examination of the differences in nurse mental health across nurse demographic and workplace characteristics before and after COVID-19 was declared a pandemic. The study findings will better inform the allocation of mental health supports and resources towards the nursing groups in greatest need of mental health interventions.

## Literature review

The prevalence of mental health problems has been increasing among nurses. A recent Statistics Canada report of 18,000 healthcare workers found 75% of nurses reported worsening mental health since March 2020—a rate higher than those of other healthcare professionals (ranging from 63% among personal support workers to 73% among physicians) (Statistics Canada, 2021).

A national pre-pandemic survey of Canadian nurses showed 48% suffered from one mental health problem with depression (36%), anxiety (26%) and post-traumatic stress disorder (PTSD) (23%) being the most prevalent nurse mental health problems (Stelnicki & Carleton, 2020). This study found nurses in Eastern Canada (e.g. Ontario) were more likely than their peers in Western Canada (e.g. Alberta) to suffer from greater levels of mental health problems (Stelnicki & Carleton, 2020). Research with British Columbia (BC) nurses found lower rates of depression (31%), but higher rates of anxiety (29%) and PTSD (49%) among this population compared to nurses nationally (Havaei et al., 2021a). In addition, more than one third of BC nurses met the criteria for burnout (Havaei et al., 2021a).

Research evidence suggests there are differences in nurse mental health across demographic and workplace characteristics. Some earlier studies found the likelihood of PTSD, depression, and burnout are higher among younger, less experienced, and female nurses (Adriaenssens et al., 2015; Mealer et al., 2009; Shields & Wilkins, 2006). More recent evidence showed years of experience and gender were not associated with mental health problems, except for nurses aged 40–49 who were more likely than their younger counterparts aged 19–29 to screen positive for any mental health problem (Stelnicki & Carleton, 2020). Professional designation, however, was significantly associated, with licensed practical nurses (LPNs) more likely to report adverse mental health compared to their registered and registered psychiatric colleagues (Stelnicki & Carleton, 2020).

While a plethora of research evidence shows nursing workplace conditions have a notable influence on nurses’ mental health, few research studies have examined the impact of health authority, healthcare sector and geographical region on nurses’ mental health. In Canada, health authorities are responsible for governing, planning, prioritizing, budgeting and allocating funds to health agencies and delivering and managing health care services within predetermined geographic regions. Each health authority has its own unique context (e.g. policies, resources) based on the populations and geographic locations they serve (Yoder-Wise et al., 2019). Owing to these differences, it is important to determine if the state of nurse mental health varies across health authorities. This is especially important considering the disproportionate impact of the COVID-19 pandemic on specific population groups and communities (Garcia et al., 2021; Jenkins et al., 2021).

The findings are mixed with respect to other workplace characteristics. While Stelnicki and Carleton did not find any association between nurse mental health and healthcare sector and geographical region pre-pandemic (Stelnicki & Carleton, 2020), Havaei et al. (2021c) found that the mental health of long-term care nurses was more negatively impacted than their peers in acute care and community care sectors during the pandemic (Havaei, Smith, Oudyk, et al., 2021c). Among other factors, this finding was attributed to the high mortality rates in long-term care homes, accounting for nearly 80% of COVID-19 deaths in Canada (Canadian Institute for Health Information, 2020). Gaining a more nuanced understanding of nurse mental health is critical to better inform provincial policy, practice and resource allocation during the ongoing pandemic.

## Methods and procedures

### Data collection, setting and sample

This secondary analysis used data from two studies conducted within one Canadian province, British Columbia (BC). In BC, health services are delivered by six health authorities, a provincial health authority that provides province-wide specialized healthcare services to the entire BC population, and a First Nation's Health Authority that is independent of the other health authorities in the province (*Health Authority: Overview*, 202*1*). In the larger studies, all nurse members (∼48,000) of the provincial nurses’ union (British Columbia Nurses Union [BCNU]) were invited to complete cross-sectional electronic surveys at two timepoints: T1, September to October 2019 (pre-pandemic); T2, June to July 2020 (during the pandemic). Survey invitations were distributed through e-mail, and participation was encouraged through follow-up email reminders, advertisement through print and BCNU social media, and raffle draws for various incentives. Participants were fully informed that participation was voluntary, and that responses were strictly confidential. A total of 5,512 and 4,523 survey responses were received for timepoints 1 and 2 respectively, yielding response rates of approximately 10% at each timepoint. Nurse classifications included registered nurses (RNs), registered psychiatric nurses (RPNs) and LPNs. For this secondary study, we included only actively working nurses that fully completed the mental health measures within each survey. To ensure participants’ anonymity, First Nation's Health Authority was excluded from our analysis due to small number of participants from this Health Authority (n < 10 at both timepoints). Given the inclusion criteria, this study analyzed data from 3,977 and 2,843 nurses across Time 1 and Time 2 respectively. Ethics approval was obtained from the participating University's Behavioral Research Ethics Board (Time 1: H18-02724; Time 2: H20-01861)

### Measures

#### Outcome variables

Four mental health screening instruments were included in both surveys. Anxiety was assessed using the seven-item Generalized Anxiety Disorder instrument (GAD-7) (Spitzer et al., 2006), depression was assessed using the nine-item Patient Health Questionnaire (PHQ-9) (Kroenke et al., 2001), post-traumatic stress disorder with the Posttraumatic Stress Symptoms instrument (PTSS-14) (Twigg et al., 2008), and burnout with the three-subscale Maslach Burnout Inventory – Human Services Survey (MBI-HSS) (Maslach et al., 1996). Based on prior research, sum score cutoffs were used to identify positive screenings. A GAD-7 score of 10 or greater indicated moderate to severe anxiety (Spitzer et al., 2006); a PHQ-9 score of 10 or greater indicated moderate to severe depression (Kroenke et al., 2001); and a PTSS-14 score of 45 or greater indicated a positive screening for PTSD (Twigg et al., 2008). For screening burnout, score cutoffs were first applied to each subscale: high emotional exhaustion was indicated by a subscale score of 27 and higher, high depersonalization by 13 or higher, low personal accomplishment by 31 or lower (Maslach et al., 1996). High emotional exhaustion and high depersonalization, or high emotional exhaustion and low personal accomplishment indicated burnout (Havaei et al., 2021a).

#### Predictors

Nurse demographic characteristics included age, designation, gender, employment status, education, experience, and role. Participants’ continuous responses to the age and experience questions were recoded into five-point ordinal categories. For several characteristics, response categories were collapsed into dichotomous variables. For designation, RNs and RPNs were compared against LPNs; for role, direct care providers were compared to leaders and educators. Workplace characteristics included workplace sector (acute care, community care, long term care), geographical region (urban, sub-urban, rural), and health authority (Fraser, Interior, Northern, Providence, Provincial, Vancouver Coastal, Vancouver Island and First Nation's Health Authorities). As indicated above, to ensure participants’ anonymity, responses from the First Nation's Health Authority were excluded from the analysis due to their small group size.

### Analysis

Statistical analyzes included descriptive statistics and regression, and were conducted using R. The prevalence of each mental health problem was obtained across demographic and workplace characteristics for each survey timepoint. To assess associations between nurses’ mental health and the predictors in a comprehensive model, an outcome variable of ‘total number of positive screenings’ was created per respondent, with values ranging from 0 (no positive screenings across GAD-7, PHQ-9, PTSS-14, MBI-HSS) to 4 (positive screenings across all four inventories). The prevalence of total positive screenings was also calculated across demographic and workplace characteristics at both survey timepoints.

Additionally, the regression models were fit using the polr function of the modern applied statistics with S package for R. Ordinal logistic regression analyses were used, specifically the proportional odds model, to examine whether demographic and workplace characteristics were significant predictors of nurses’ positive screenings on mental health assessments. Odds ratios and 95% confidence intervals were computed. For most predictors, the group with the lowest dependent variable means in the first timepoint were used as the reference category for ease of interpretation of odds ratios. Multicollinearity was evaluated using variance inflation factor values, which suggested that there was high collinearity between age and experience variables. Thus, the experience variable was excluded from the final regression model, leaving nine predictors in the model. The final regression model included six demographic (age, designation, gender, employment status, education and role) and three workplace characteristics (sector, geographical region and health authority).

## Results

Proportions of positive screenings for each mental health outcome by demographic and workplace characteristic categories are presented in Table 1. The overall trend in proportions between timepoints indicates that positive screening proportions increased between Time 1 and Time 2, across all four mental health outcomes. For example, positive screening proportions increased across all four instruments for both genders: for the depression measure, 31% of female respondents met the criteria for depression in Time 1 compared to 42% in Time 2; similarly for males, 30% in Time 1 compared to 39% in Time 2. The trend of increasing positive screening proportions across timepoints was consistent across every level of each demographic and workplace characteristic for depression, anxiety, and PTSD, with the sole exception of PTSD in nurses casually employed where there was a subtle decline in positive screenings across timepoints (46% in Time 1, to 45% in Time 2).

**Table 1.** Proportions of Positive Screenings for Each Mental Health Problem by Individual and Workplace Characteristic Categories*.*

Characteristic	Time 1				Time 2			
	PHQ9^a^	GAD7^b^	PTSS14^c^	MBI-HSS^d^	PHQ9^a^	GAD7^b^	PTSS14^c^	MBI-HSS^d^
	% (n)	% (n)	% (n)	% (n)	% (n)	% (n)	% (n)	% (n)
Demographic characteristics								
*Age*								
Under 25	39.2 (71)	45.3 (82)	55.2 (100)	43.1 (78)	43.6 (44)	52.5 (53)	59.4 (60)	56.4 (57)
25 to 34	33.8 (458)	30.9 (419)	52.3 (708)	44.4 (601)	44.9 (353)	45.2 (355)	55.2 (434)	43.9 (345)
35 to 44	31.3 (318)	28.1 (285)	50.4 (512)	35.1 (356)	46.6 (361)	40.8 (316)	56.3 (436)	39.1 (303)
45 to 54	28.7 (239)	25.9 (216)	44.8 (374)	28.9 (241)	40.4 (271)	35.6 (239)	50.2 (337)	28.6 (192)
55 and above	26.5 (157)	20.7 (123)	39.5 (234)	21.9 (130)	33.3 (170)	26.2 (134)	43.1 (220)	23.7 (121)
*Designation*								
RN and/or RPN	29.9 (1,006)	27.7 (933)	47.8 (1,610)	35.2 (1,186)	42.2 (987)	38.7 (905)	52 (1,216)	35.7 (834)
LPN	38.7 (237)	31.4 (192)	52 (318)	35.9 (220)	41.9 (212)	37.9 (192)	53.6 (271)	36.4 (184)
*Gender*								
Female	31.3 (1,141)	28.8 (1,047)	48.7 (1,773)	35.1 (1,276)	42.4 (1,133)	38.9 (1,041)	52.4 (1,401)	35.5 (948)
Male	30.3 (102)	23.1 (78)	46 (155)	38.6 (130)	39.1 (66)	33.1 (56)	50.9 (86)	41.4 (70)
*Education*								
Diploma/certificate	33.8 (400)	27.4 (324)	46.7 (553)	32.6 (386)	39.8 (378)	35.5 (337)	49.7 (472)	32.4 (308)
Undergraduate degree	29.9 (593)	29.3 (582)	49.8 (989)	38.3 (760)	43.1 (579)	40.5 (543)	54.5 (731)	39.5 (530)
Graduate degree	30.9 (250)	27 (219)	47.7 (386)	32.1 (260)	43.9 (242)	39.4 (217)	51.5 (284)	32.7 (180)
*Employment status*								
Casual	27.5 (117)	26.1 (111)	45.9 (195)	33.6 (143)	35.5 (99)	35.5 (99)	44.8 (125)	28.7 (80)
Part-time	29 (310)	26.2 (280)	46.3 (495)	32.6 (349)	38.8 (320)	37 (305)	50.1 (413)	33.2 (274)
Full-time	32.9 (816)	29.6 (734)	49.9 (1,238)	36.8 (914)	44.9 (780)	39.9 (693)	54.6 (949)	38.2 (664)
*Experience*								
5 years or less	35.4 (428)	33.6 (407)	52.6 (636)	42.2 (511)	44.2 (316)	45.5 (325)	56.5 (404)	43.1 (308)
6 to 10 years	32.8 (285)	28.5 (247)	53 (460)	40.8 (354)	47.1 (274)	41.8 (243)	55 (320)	40.4 (235)
11 to 15 years	30.4 (207)	28.1 (191)	48.7 (331)	35.3 (240)	43.4 (218)	38.2 (192)	52.6 (264)	36.3 (182)
16 to 20 years	26.8 (86)	24 (77)	45.8 (147)	31.2 (100)	41.5 (114)	34.9 (96)	53.8 (148)	35.6 (98)
21 years or more	26.4 (237)	22.6 (203)	39.4 (354)	22.4 (201)	36 (277)	31.3 (241)	45.6 (351)	25.4 (195)
*Role*								
Direct care provider	31.5 (1,128)	28.5 (1,018)	49.3 (1,764)	36.1 (1,292)	42.1 (1,030)	39 (952)	53.1 (1,297)	36.2 (884)
Leader or educator	28.8 (115)	26.8 (107)	41.1 (164)	28.6 (114)	42.4 (169)	36.3 (145)	47.6 (190)	33.6 (134)
Workplace characteristics								
*Sector*								
Acute care	31.3 (932)	28.3 (844)	50.1 (1,493)	37.7 (1,122)	42.4 (771)	39.8 (723)	54.1 (983)	37.9 (689)
Community care	30.4 (207)	28.9 (197)	44.9 (306)	26 (177)	43 (293)	37.6 (256)	49.6 (338)	31.3 (213)
Long-term care	32.9 (104)	26.6 (84)	40.8 (129)	33.9 (107)	39.1 (135)	34.2 (118)	48.1 (166)	33.6 (116)
*Geographical region*								
Urban	30.3 (752)	28.2 (699)	47.8 (1,184)	34.3 (850)	43 (772)	39 (700)	52.8 (948)	36.4 (653)
Suburban	30.4 (215)	27.9 (197)	48.9 (346)	36.2 (256)	38.5 (214)	37.9 (211)	50.4 (280)	34.5 (192)
Rural	34.9 (276)	29 (229)	50.3 (398)	37.9 (300)	43.2 (213)	37.7 (186)	52.5 (259)	35.1 (173)
*Health authority*								
Fraser	29.4 (271)	27.5 (254)	46.7 (431)	36.9 (340)	42.3 (285)	38.7 (261)	53.6 (361)	36.4 (245)
Interior	33.7 (247)	30.8 (226)	50.1 (368)	38 (279)	43.8 (213)	40.1 (195)	56 (272)	42 (204)
Northern	33.8 (153)	29.1 (132)	51 (231)	35.8 (162)	44.4 (91)	45.4 (93)	52.2 (107)	34.6 (71)
Providence	23.5 (31)	22.7 (30)	41.7 (55)	34.1 (45)	53.3 (56)	48.6 (51)	63.8 (67)	44.8 (47)
Provincial	26.9 (105)	27.7 (108)	45.1 (176)	23.3 (91)	35.5 (60)	33.1 (56)	46.2 (78)	26 (44)
Vancouver Coastal	28.1 (176)	24.3 (152)	43.3 (271)	34 (213)	37.4 (239)	33.2 (212)	44.8 (286)	29.9 (191)
Vancouver Island	36.1 (260)	31 (223)	55 (396)	38.3 (276)	45.1 (255)	40.5 (229)	55.9 (316)	38.2 (216)

*Note*. ^a^ Anxiety screening tool; ^b^ Depression screening tool; ^c^ PTSD screening tool; ^d^ Burnout screening tool. Time 1, pre-COVID-19; Time 2, during COVID-19.

For burnout screenings, several groups within characteristics maintained consistent proportions or slight decreases across timepoints. For demographic characteristics, these groups consisted of respondents aged 25 to 34 (44% T1 to 44% T2), respondents aged 45 to 54 (29% T1 to 29% T2), respondents with diploma or certificate as their highest level of education (33% T1 to 32% T2), and those casually employed (34% T1 to 29% T2). For workplace characteristics, stable or improving groups were long-term care (34% T1 to 34% T2), suburban (36% to 35%), and rural (38% to 35%). For health authorities, Vancouver Island (38% to 38%), Vancouver Coastal (34% to 30%), Northern (36% to 35%), and Fraser (37% to 36%) had stable or decreasing proportions of nurses screening positive for burnout. All other groups had rising proportions across timepoints, with the greatest increases occurring in the groups for respondents under 25 (43% to 56%; + 13.3% change), Providence Health authority (34% to 45%; + 10.7% change), and community care sector (26% to 31%; + 5.3% change).

Proportions for number of positive screenings are presented in Table 2. Mirroring the overall trend in Table 1, the proportions of respondents that had zero or one positive screenings generally decreased between timepoints across all demographic and workplace characteristics, and proportions for three or four positive screenings (poor mental health indicated by three or all four instruments) increased between timepoints. The groups with the greatest increases in proportions for three or four positive screenings were Providence Health authority ( + 28.5%), respondents aged 35 to 44 (11.7%), respondents with undergraduate degrees as highest level of education ( + 9.9%) and working in community care sector ( + 9.6%), and urban settings ( + 9.5%).

**Table 2.** Proportions of Nurses Screening Positive for Zero, One, Two, Three, or Four Mental Health Problems at Time 1 and Time 2.

Characteristic	Number of positive screenings, % (n)									
	Time 1					Time 2				
	0	1	2	3	4	0	1	2	3	4
Demographic characteristics										
*Age*										
Under 25	32 (58)	13.8 (25)	13.8 (25)	19.9 (36)	20.4 (37)	19.8 (20)	17.8 (18)	18.8 (19)	17.8 (18)	25.7 (26)
25 to 34	34.1 (462)	18.5 (250)	15.7 (213)	15.2 (206)	16.5 (223)	29.3 (230)	15.5 (122)	15.3 (120)	16.7 (131)	23.3 (183)
35 to 44	38.3 (389)	19.5 (198)	14.2 (144)	14.9 (151)	13.1 (133)	31.9 (247)	15.5 (120)	12.9 (100)	17.1 (132)	22.6 (175)
45 to 54	43 (359)	20.7 (173)	12.7 (106)	11.9 (99)	11.6 (97)	37.4 (251)	18.5 (124)	11.3 (76)	17.4 (117)	15.4 (103)
55 and above	51.3 (304)	18 (107)	9.8 (58)	12.6 (75)	8.3 (49)	47 (240)	15.7 (80)	11.7 (60)	15.5 (79)	10.2 (52)
*Designation*										
RN and/or RPN	40.1 (1,350)	18.8 (632)	14.2 (478)	14.1 (473)	12.8 (432)	35.2 (822)	15.6 (365)	13.3 (310)	17.2 (403)	18.7 (437)
LPN	36.3 (222)	19.8 (121)	11.1 (68)	15.4 (94)	17.5 (107)	32.8 (166)	19.6 (99)	12.8 (65)	14.6 (74)	20.2 (102)
*Gender*										
Female	39.3 (1,429)	19 (692)	13.8 (502)	14.5 (527)	13.5 (490)	34.6 (925)	16.4 (438)	13.2 (354)	16.9 (451)	18.9 (506)
Male	42.4 (143)	18.1 (61)	13.1 (44)	11.9 (40)	14.5 (49)	37.3 (63)	15.4 (26)	12.4 (21)	15.4 (26)	19.5 (33)
*Education*										
Diploma/certificate	41.4 (490)	18.5 (219)	12.2 (144)	13.9 (164)	14 (166)	38.2 (363)	16.6 (158)	12.1 (115)	15.7 (149)	17.4 (165)
Undergraduate degree	38 (754)	18.9 (375)	15 (297)	14 (277)	14.2 (281)	32.3 (434)	15.9 (214)	13.6 (183)	18 (241)	20.1 (270)
Graduate degree	40.5 (328)	19.6 (159)	13 (105)	15.6 (126)	11.4 (92)	34.7 (191)	16.7 (92)	14 (77)	15.8 (87)	18.9 (104)
*Employment status*										
Casual	42.1 (179)	20.5 (87)	12 (51)	12.9 (55)	12.5 (53)	41.9 (117)	15.8 (44)	13.3 (37)	14 (39)	15.1 (42)
Part-time	42.2 (452)	18.6 (199)	13.8 (148)	13.6 (145)	11.8 (126)	37.5 (309)	15.4 (127)	14.3 (118)	16.4 (135)	16.5 (136)
Full-time	37.9 (941)	18.8 (467)	14 (347)	14.8 (367)	14.5 (360)	32.3 (562)	16.8 (293)	12.7 (220)	17.4 (303)	20.8 (361)
*Experience*										
5 years or less	34 (411)	17.6 (213)	15.8 (191)	16 (193)	16.7 (202)	29.1 (208)	14.8 (106)	17.2 (123)	15.5 (111)	23.4 (167)
6 to 10 years	34.9 (303)	19.9 (173)	15 (130)	15.6 (135)	14.6 (127)	31.4 (183)	16.5 (96)	10.7 (62)	19.2 (112)	22.2 (129)
11 to 15 years	38.4 (261)	21.2 (144)	13.5 (92)	13.4 (91)	13.5 (92)	33.3 (167)	18.7 (94)	12.4 (62)	15.5 (78)	20.1 (101)
16 to 20 years	43 (138)	21.5 (69)	11.5 (37)	12.8 (41)	11.2 (36)	34.9 (96)	17.5 (48)	12.4 (34)	17.5 (48)	17.8 (49)
21 years or more	51.1 (459)	17.1 (154)	10.7 (96)	11.9 (107)	9.1 (82)	43.4 (334)	15.6 (120)	12.2 (94)	16.6 (128)	12.1 (93)
*Role*										
Direct care provider	38.7 (1,383)	19.2 (686)	14.1 (506)	14.2 (508)	13.8 (495)	34.3 (838)	16.3 (399)	13.4 (327)	16.8 (410)	19.2 (470)
Leader or educator	47.4 (189)	16.8 (67)	10 (40)	14.8 (59)	11 (44)	37.6 (150)	16.3 (65)	12 (48)	16.8 (67)	17.3 (69)
Workplace characteristics										
*Sector*										
Acute care	38.1 (1,135)	18.8 (561)	14.7 (439)	14.4 (428)	14 (417)	33.1 (602)	16.1 (293)	14.1 (256)	16.7 (303)	20 (363)
Community care	43.8 (298)	19.5 (133)	11.2 (76)	13.8 (94)	11.7 (80)	37.4 (255)	16 (109)	11.5 (78)	17.8 (121)	17.3 (118)
Long-term care	44 (139)	18.7 (59)	9.8 (31)	14.2 (45)	13.3 (42)	38 (131)	18 (62)	11.9 (41)	15.4 (53)	16.8 (58)
*Geographical region*										
Urban	40.3 (999)	19.5 (483)	13.2 (328)	13.3 (330)	13.7 (339)	34.3 (616)	16.2 (291)	12.9 (231)	16.9 (304)	19.6 (352)
Suburban	39.3 (278)	19.2 (136)	12.9 (91)	15.8 (112)	12.7 (90)	36.9 (205)	16.2 (90)	13.1 (73)	16.4 (91)	17.4 (97)
Rural	37.3 (295)	16.9 (134)	16.1 (127)	15.8 (125)	13.9 (110)	33.9 (167)	16.8 (83)	14.4 (71)	16.6 (82)	18.3 (90)
*Health authority*										
Fraser	39.8 (367)	19.1 (176)	14.8 (136)	13.4 (124)	12.9 (119)	33.1 (223)	17.4 (117)	13.9 (94)	16.8 (113)	18.8 (127)
Interior	38.3 (281)	17.7 (130)	12.5 (92)	16.1 (118)	15.4 (113)	31.7 (154)	15.6 (76)	14.2 (69)	16 (78)	22.4 (109)
Northern	37.3 (169)	20.3 (92)	12.8 (58)	14.6 (66)	15 (68)	33.2 (68)	15.6 (32)	12.7 (26)	18.5 (38)	20 (41)
Providence	46.2 (61)	18.2 (24)	13.6 (18)	11.4 (15)	10.6 (14)	22.9 (24)	18.1 (19)	8.6 (9)	26.7 (28)	23.8 (25)
Provincial	45.1 (176)	21.3 (83)	10.8 (42)	11 (43)	11.8 (46)	44.4 (75)	12.4 (21)	16 (27)	12.4 (21)	14.8 (25)
Vancouver Coastal	42.3 (265)	20.8 (130)	12.8 (80)	13.1 (82)	11 (69)	41.9 (268)	15.5 (99)	12.8 (82)	14.9 (95)	14.9 (95)
Vancouver Island	35.1 (253)	16.4 (118)	16.7 (120)	16.5 (119)	15.3 (110)	31.2 (176)	17.7 (100)	12 (68)	18.4 (104)	20.7 (117)

*Note*. For number of positive screenings, 0 = respondent did not screen positive across any of the four mental health problems; 1 = respondent screened positive for one mental health problem; 2 = screened positive for two problems; 3 = screened positive for three problems; 4 = screened positive for all four assessed problems. Time 1, pre-COVID-19; Time 2, during COVID-19.

Odds ratios and 95% confidence intervals of the ordinal logistic regression model are shown in Table 3. For demographic characteristics, no significant differences were found in the likelihood of positive mental health screenings across participants of differing gender, education, and role (compared to the reference groups) in both timepoints. Significant differences were, however, found within age groups for both timepoints, with younger groups sequentially more likely to have greater number of positive mental health screenings. Compared to the 55 and above age group, odds ratios indicated that respondents 45 to 54 were 1.3 times more likely to have more positive screenings (Time 1 OR 1.32, 95% CI: 1.08, 1.61; Time 2 OR 1.33, 95% CI: 1.07, 1.64), while respondents under 25 were 2.5 times more likely (Time 1 OR 2.54, 95% CI: 1.84, 3.49; Time 2 OR 2.52, 95% CI: 1.7, 3.73). Within designations, LPNs were significantly more likely to have more positive mental health screenings than their registered nurse colleagues (i.e. RNs and/or RPNs) in Time 1 (OR 1.25, 95% CI: 1.02, 1.54), but not in Time 2. Within employment status, respondents working full-time were more likely to have more positive screenings than casual employees for both timepoints (Time 1 OR 1.37, 95% CI: 1.13, 1.66; Time 2 OR 1.62, 95% CI: 1.28, 2.05). Respondents working part-time were not significantly different in their odds of having more positive screenings than those working casually.

**Table 3.** Odds Ratios from Ordinal Logistic Regression Models Predicting Number of Positive Screenings on Mental Health Measures by Demographic and Workplace Characteristics, at Time 1 and Time 2.

Characteristic	Odds ratio (95% CI)	
	Time 1	Time 2
Demographic characteristics		
*Age*		
Under 25	2.54 (1.84, 3.49)***	2.52 (1.7, 3.73)***
25 to 34	2.05 (1.68, 2.51)***	1.99 (1.59, 2.49)***
35 to 44	1.66 (1.36, 2.03)***	1.85 (1.49, 2.3)***
45 to 54	1.32 (1.08, 1.61)**	1.33 (1.07, 1.64)**
55 and above	1.00	1.00
*Designation*		
RN and/or RPN	1.00	1.00
LPN	1.25 (1.02, 1.54)*	1.05 (0.84, 1.32)
*Gender*		
Female	1.05 (0.85, 1.3)	1.08 (0.81, 1.44)
Male	1.00	1.00
*Employment status*		
Casual	1.00	1.00
Part-time	1.11 (0.9, 1.37)	1.28 (0.99, 1.65)
Full-time	1.37 (1.13, 1.66)**	1.62 (1.28, 2.05)***
*Education*		
Diploma/certificate	1.11 (0.92, 1.35)	1.01 (0.81, 1.25)
Undergraduate degree	1.08 (0.93, 1.25)	1.07 (0.89, 1.28)
Graduate degree	1.00	1.00
*Role*		
Direct care provider	1.15 (0.94, 1.4)	1.08 (0.89, 1.32)
Leader or educator	1.00	1.00
Workplace characteristics		
*Sector*		
Acute care	1.2 (0.94, 1.52)	1.06 (0.84, 1.33)
Community care	1.11 (0.85, 1.45)	1.05 (0.82, 1.34)
Long-term care	1.00	1.00
*Geographical region*		
Urban	1.00	1.00
Suburban	0.99 (0.85, 1.16)	0.89 (0.74, 1.07)
Rural	1.03 (0.87, 1.22)	0.95 (0.78, 1.16)
*Health authority*		
Fraser	1.31 (1.05, 1.65)*	1.48 (1.08, 2.02)*
Interior	1.52 (1.2, 1.93)***	1.69 (1.21, 2.35)**
Northern	1.34 (1.03, 1.75)*	1.49 (1.02, 2.2)*
Providence	1.01 (0.7, 1.45)	2.18 (1.41, 3.39)***
Provincial	1.00	1.00
Vancouver Coastal	1.18 (0.93, 1.49)	1.08 (0.79, 1.48)
Vancouver Island	1.7 (1.35, 2.14)***	1.68 (1.22, 2.31)**

*Note.* Characteristic groups with an odds ratio of 1.00 are the reference groups for that characteristic. The nurse characteristic *experience* was excluded from the regression model due to potential high collinearity with *age*.

*p < 0.05, **p < 0.01, ***p < 0.001 – Group is statistically significantly different from reference group. Time 1, pre-COVID-19; Time 2, during COVID-19.

For workplace characteristics, no significant differences were found in the likelihood of positive mental health screenings across participants of varying sectors and geographical regions (compared to the reference groups). For health authority, Provincial Health Services Authority served as the reference group, having lower odds of more positive screenings than the other health authorities for both timepoints. Vancouver Coastal Health was not significantly different from the reference group across both timepoints. In Time 1, Providence Health also was not significantly different from the reference, while the remainder of the health authorities had higher odds of respondents having more positive screenings, ranging from 1.3 times more likely for Fraser Health (OR 1.31, 95% CI: 1.05, 1.65) to 1.7 times more likely for Vancouver Island (OR 1.7, 95% CI: 1.35, 2.14). In Time 2, Vancouver Island remained stable in relation to the reference (OR 1.68, 95% CI: 1.22, 2.31), while Fraser Health, Interior Health, and Northern Health continued to indicate greater odds of more positive mental health screenings among their nursing employees than the reference and saw slight increases relative to their respective Time 1 odds ratios. Providence Health, which had no significant difference in odds from the reference in Time 1, was more than twice as likely to have more positive screenings among their nursing staff than Provincial Health in Time 2 (OR 2.18, 95% CI: 1.41, 3.39), and had the largest Time 2 odds ratio out of all the health authorities.

## Discussion

Between Time 1 and Time 2, key study findings were increases in positive screenings for males and females across all four mental health outcomes. These findings are similar to evidence emerging globally of significant psychological threats to care providers, including nurses (World Health Organization, 2020). Many countries affected by COVID-19 have identified the same psychological risks to healthcare providers. The World Health Organization formally recognized COVID-19 stressors affecting the physical and mental health of providers—namely anxiety and depression followed by burnout and PTSD (World Health Organization, 2020). For example, Italian psychologists studied the impact of COVID-19 on healthcare providers in a country initially overwhelmed with high COVID-19 mortality rates (Barello et al., 2020). They found that psycho-somatic symptoms and burnout increased in relation to increased job demands and ongoing exposure to others’ emotional distress and moral suffering (Barello et al., 2020). These researchers urged healthcare leaders to recognize and mitigate sources of work-related stress within healthcare settings; and to develop collaborative and targeted approaches with workers to address their specific concerns and support needs (Barello et al., 2020).

One important workplace strategy to address nurses’ mental health needs are regular self-assessments using simple and publicly available mental health screening tools such as GAD-7 and PHQ-9 (Kang et al., 2020). Personal check-ins with validated screening tools may offer healthcare providers a greater sense of control and objective evidence for reaching out for professional help (Kang et al., 2020). Self-monitoring is an effective strategy to offer more assurance to healthcare workers (Cullen et al., 2020), but healthcare organizations and leaders must be prepared to follow through with appropriate mental health supports for healthcare workers, especially during widespread crisis (Barello et al., 2020). While most publicly employed healthcare providers in Canada have access to employee assistance programs that offer free access to counselling services, access is often inadequate and compromised by mental health stigma (Havaei et al., 2021a). In BC, nurses’ access is limited to three to four counselling sessions for each mental health issue; raising concerns about adequate management of serious mental health problems. Therefore, it is essential and timely for occupational health and safety services, such as the employee assistance programs, to be revisited and adapted to better meet rising mental health needs of nurses and other healthcare workers during the COVID-19 pandemic.

Workload management and access to adequate protective equipment are other evidence-based strategies for reducing mental duress on healthcare providers—and more easily said than done—especially with finite human resources (Pfefferbaum & North, 2020). Healthcare organizations and leadership, however, do have low-cost options known as leader empowering behaviors or psychological empowerment strategies (Cziraki et al., 2020). Leader empowerment strategies reinforce the meaning of work and workers’ valued contributions. These basic strategies, delivered via leaders or through peer-to-peer supports, can prevent and limit burnout and other stress-related outcomes by creating collaborative conditions that promote well-being and retention (Cziraki et al., 2020).

Another key study finding was identification of one health authority, Providence Health Authority, with significantly higher proportions of nurse mental health problems than the other health authorities during the pandemic. Providence oversees care delivery for vulnerable populations in inner city Vancouver, including high proportions of patients who are homeless with mental health and substance use disorders. COVID-19 has increased the need for post-traumatic stress-related psychological care for these populations given significant barriers for medication and treatment access during this time. During the pandemic, there has also been a worsening opioid crisis in Canada further requiring nurses and other care providers to adapt quickly to reach vulnerable populations and maintain critical “whole person” services, challenging in the best of times (Kanzler & Ogbeide, 2020). It is not surprising, therefore, that nurses working in this particular health authority are reporting increasing mental health problems during the pandemic.

Two final study findings of importance were higher proportions of positive screenings for LPNs versus RNs and RPNs and for full-time versus part-time and casual employees. For both findings, a common denominator was under-staffing and increased workloads due to COVID-19 (Beckett et al., 2021). Due to nursing shortages, care delivery has focused on patients with highly acute care needs. Since LPNs are typically assigned to care for stable, non-complex patients with support from RN team members, excess workloads and sicker patients during COVID-19 have put undue pressure on LPNs to provide patient care beyond their professional (even legal) capacity. Similarly, the nursing shortage has resulted in full-time nurses being asked to work extra shifts and to work overtime (in some cases in a mandatory manner) without the necessary rest periods and work gaps to recover from workplace stressors. One survey study in the US found that nurses working more than 40 h weekly and or missing 30-min rest breaks experienced more fatigue and sleep disturbances and greater self-reports of mental health problems during the pandemic (Sagherian et al., 2020). Research on PTSD in nurses has established that persistent exposure to traumatic events, including patients’ physical and emotional distress, can accumulate over time—with more cumulative exposure in full-time exposure than nurses working less hours (Schuster & Dwyer, 2020).

### Strengths and limitations

This is the first Canadian study examining the differences in nurses’ mental health needs across demographic and workplace characteristics using pre- and during COVID-19 data. Additionally, to our knowledge, no other study has analyzed nurse mental health data at a health authority level in Canada. Despite these strengths, there are also some limitations including low response rates and potential non-response bias. Owing to these limitations, the study findings should be cautiously generalized to other contexts and samples including non-active and non-unionized nurses. Second, it is highly possible that this study has underestimated the prevalence of nurse mental health problems due to social desirability bias and also because nurses with most significant mental health problems likely were unable to participate in the surveys. Future research should use more sophisticated research methods that facilitate obtaining a representative sample of the nursing workforce and minimize social desirability biases. Finally, due to the cross-sectional nature of the surveys, we caution readers against establishing cause-and-effect conclusions.

## Conclusion

Our study has shown that more granular data on nurse demographics and workplace characteristics can inform healthcare organizations and leaders of where mental health resources and supports may be most needed. We have also provided some evidence-based strategies for addressing nurses’ mental health during the COVID-19 pandemic. We believe many structural and psychological strategies we have reported from the literature are universals that should be in place, proactively and preventively, to buffer nurses against workplace challenges that are likely to increase during the COVID-19 crisis.
